# Changes in Serum Natriuretic Peptide Levels after Percutaneous Closure of Small to Moderate Ventricular Septal Defects

**DOI:** 10.1100/2012/328697

**Published:** 2012-04-26

**Authors:** Yuksel Kaya, Ramazan Akdemir, Huseyin Gunduz, Sani Murat, Orhan Bulut, İbrahim Kocayigit, M. Bulent Vatan, M. Akif Cakar, Ekrem Yeter, Harun Kilic, Mustafa Tarik Agac, Zeydin Acar

**Affiliations:** ^1^Department of Cardiology, Van Yüksek Ihtisas Hospital, 65200 Van, Turkey; ^2^Department of Cardiology, Faculty of Medicine, Sakarya University, Esentepe, 54054 Sakarya, Turkey; ^3^Department of Cardiology, Research and Education Hospital, Dıskapi/Etlik Ihtisas, 06110 Ankara, Turkey; ^4^Department of Cardiology, Ahi Evren Heart and Vascular Surgery Training and Research Hospital, 61040 Trabzon, Turkey

## Abstract

*Background*. B-type natriuretic peptide has been shown to be a very sensitive and specific marker of heart failure. In this study, we aimed to investigate the effect of percutaneous closure of ventricular septal defects with Amplatzer septal occluders on brain natriuretic peptide levels. *Methods*. Between 2008 and 2011, 23 patients underwent successfully percutaneous ventricular septal defect closure in 4 cardiology centers. Brain natriuretic peptide levels were measured in nine patients (4 male, mean ages were 25.3 ± 14.3) who underwent percutaneous closure with Amplatzer occluders for membranous or muscular ventricular septal defects were enrolled in the study. Brain natriuretic peptide levels were measured one day before and one month after the closure. Patients were evaluated clinically and by echocardiography one month after the procedure. *Results*. Percutaneous closures of ventricular septal defects were successfully performed in all patients. There was not any significant adverse event in patients group during followup. Decrease in brain natriuretic peptide levels after closure were statistically significant (97.3 ± 78.6 versus 26.8 ± 15.6, *P* = 0.013). *Conclusion*. Brain Natriuretic Peptide levels are elevated in patients with ventricular septal defects as compared to controls. Percutaneous closure of Ventricular Septal Defect with Amplatzer occluders decreases the BNP levels.

## 1. Introduction

Ventricular Septal Defect (VSD) is the most common congenital heart defect and seen in approximately 30% of all congenital heart lesions [1–5]. Up to 70% of these VSDs are localized in perimembranous region, and most of these defects require invasive treatment due to the heart failure, left heart volume overload, evolving aortic regurgitation, or following endocarditis [1, 6–11]. Surgery is still standard treatment, but this procedure is more invasive than percutaneous closure and requires cardiopulmonary bypass and cardioplegic arrest. Surgical treatment has negative impact on quality of life of patients because of incisional trauma, limitations on activity after surgery and surgical scars [1–14]. Therefore, percutaneous closure of VSD has become an important alternative option to surgical treatment. Nowadays, most of the VSDs are increasingly being successfully closed by percutaneous approach [1–11].

Brain natriuretic peptide (BNP) is a neurohormone released by cardiac ventricles in response to volume and pressure load [4, 5, 12, 13]. These natriuretic hormones have important role on regulating extracellular fluid volume and blood pressure. BNP is a circulating mediator that has been correlated with the degree of heart failure and higher in patients with VSDs as a result of hyperkinetic circulation [4, 5, 12, 13]. In this study, we evaluated the BNP levels one day before and one month after the percutaneous closure of VSDs with Amplatzer occluders in adults and children.

## 2. Materials and Methods

Between 2008 and 2011, total of 23 patients underwent successfully percutaneous VSD closure attempt in 4 cardiology centers. BNP levels were measured in nine patients (4 male, mean age was 25.3 ± 14.3) who underwent percutaneous closure with Amplatzer occluders for membranous or muscular Ventricular Septal Defects enrolled in the study. BNP levels were measured day before and thirty days after the closure. Patients were evaluated clinically and by echocardiography one month after the procedure. Inclusion criteria: clinical and/or echocardiographic evidence of a significant left-to-right shunt through the VSD if there were one of those criteria: cardiomegaly on chest X-ray, left atrial enlargement, defined as left atrial to aortic ratio >1.5, left ventricular enlargement (left ventricular volume overload), defined as a left ventricular end-diastolic diameter ≥2 standard deviations (SD) above the average for the patient's age, symptoms including frequent respiratory infections and/or failure to thrive, and a New York Heart Association functional class II or greater. Frequent respiratory infections were defined as more than six events a year. Failure to thrive was defined according to the literature [2]. We found that patients who were ≥5 years old and ≥15 kg would be eligible for percutaneous closure of VSD. Only subjects with a rim of at least 2 mm separating the aortic valve from the VSD were included. Patients who have operated before for VSD and have significant residual shunt from the repaired area were also included. We excluded patients with VSD and prolapse of an aortic cusp and patients with VSD and misalignment. Patients with larger than 12 mm VSD and pulmonary artery pressure greater than 2/3 of systemic arterial pressure were also excluded the study.

Patients or parents of the children gave their written informed consent to the procedure. The patients' general characteristics are reported in Table 1.

A written consent was obtained from all patients, and our local ethical committee approved the study.

### 2.1. Device, Procedure, and Delivery Systems

The muscular or membranous Amplatzer VSD occluder device and Amplatzer duct occluder II device were (AGA Medical Corporation, Golden Valley, MN, USA) used for closure in all patients as was described previously [1–11, 15]. The thickness of wire for the 12 and 14 mm devices is 0.0059; the rest of devices are made from 0.0049 Nitinol wire.

Right femoral vein and artery sheaths were placed. Right and left cardiac catheterization was performed firstly. The shunt volume was calculated by echocardiographic and oximetric measurements [16]. Left ventriculography was performed in LAO cranial and left lateral projections. VSD defect size, distance to aortic and tricuspid valves, was evaluated LAO cranial and left lateral projections. The device which will be used for closing was decided using angiography and transthoracic echocardiography. The device was chosen 1-2 mm larger than the size of the VSD.

**Figure 1:**
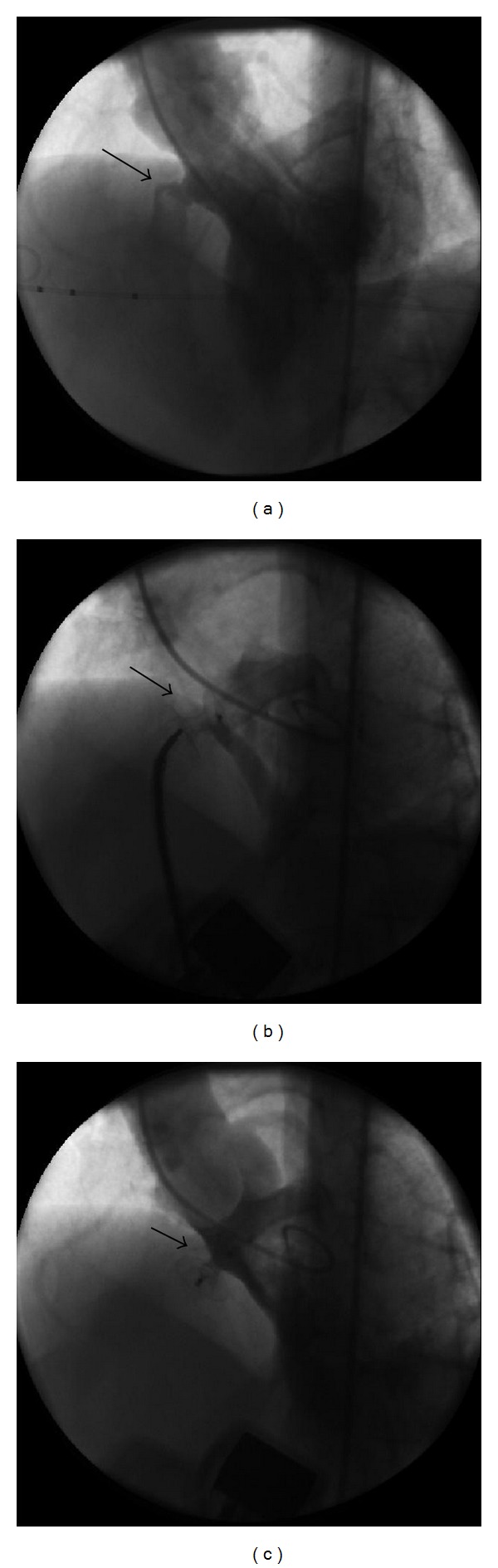
(a) Muscular VSD before the closure, (b) muscular VSD during the occluder device positioned, (c) muscular VSD successfully occluded by the device after closure.

Implantation of the VSD occluder was performed according to standard techniques previously described under the guidance of transthoracic and transesophageal echocardiography [1–11]. VSD was passed through by right Judkins' catheter via aorta, and a hydrophilic 0.035 inch wire was placed to pulmonary artery. Catheter was pushed, and wire was changed by noodle wire and it was captured by a snare in the pulmonary artery and wire pulled back from the femoral vein sheath. During the catheter was in VSD region, delivery sheath was advanced over the noodle wire and the dilator slowly drawn back, and the sheath was pushed toward the apex of the LV. The device was screwed into the cable, left side of the device opened first, and right side of the device was opened after control left ventricular angiography. If the result was satisfactory, then device was released and control angiography and echocardiography was performed and repeated 10 minute after device release (Figures 1(a), 1(b), and 1(c)). Hemostasis was achieved, and patients recovered overnight. Heparin was used in all patients to keep the activated clotting time greater than 200 sec, and antibiotic prophylaxis was given at the beginning of the case and two doses thereafter. Next day, transthoracic echocardiography, chest radiograph, and ECG were performed prior to discharge from the hospital. All patients underwent complete cardiac and laboratory evaluation after 1 month, at 6 months, and after 1 year.

BNP levels were measured one day before and one month after the percutaneous closure of VSDs with Amplatzer occluders. One month later, all patients were evaluated clinically and by echocardiography. Written informed consent was obtained in all patients.

## 3. Statistical Analysis

Statistical analysis was performed using the Statistical Package for Social Sciences (SPSS, Chicago, IL, USA), version 15.0 software for Windows. Descriptive statistics were made, and all data were expressed as mean ± standard deviation and % ratio. The quantitative values between pre- and postprocedures were compared using paired sample *t*-test. *P* value of <0.05 was considered as statistically significant in all cases.

## 4. Results

Tables 1, and 2 summarize the patients' clinical, demographic, and procedural data. Percutaneous closures of VSDs were successfully performed in all patients. The median age of the patients was 25.3 years (range 8–53 years). The median size of VSD was 7.0 mm (range 4–12) as measured by transthoracic echocardiography.

There was not any death or urgent cardiac surgery required complication. There was one device embolization which was seen in patient number 3. The embolized device was stuck in abdominal aorta, and snare retrieval was tried before but yielded unsuccessful. Device was rescrewed and replaced to the VSD defect region successfully.

Third-degree atrioventricular block was occurred in one patient, and temporary pace maker was inserted for 24 hours. Normal sinusal rhythm was restored after 12 hours monitoring, and 1 mg/kg cortisone was administered for this patient by intravenous route. Intradevice residual shunt remained in 3 patients for 3 days and residual shunt was remained in one patient. Mild groin hematoma was occurred in three patients.

There was not death, endocarditis, or device-related complication in the follow-up period. Residual shunt was remained in one patient in whom the defect was closed by ADO II and simultaneous ASD closure.

**Followup**

**Figure 2:**
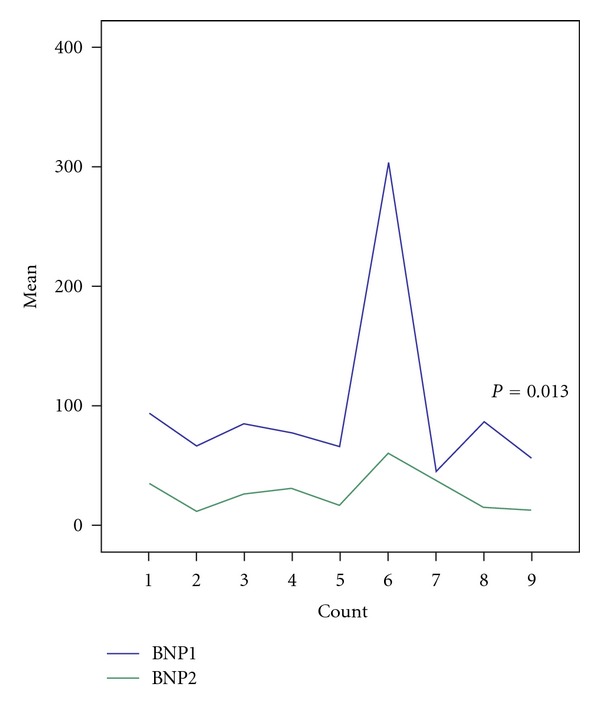
BNP levels before and after VSD closure.

Mean preprocedural BNP level obtained one day before the closure was 97.3 ± 78.6 pg/mL. BNP levels measured one month after the closure decreased in all patient compared with the preprocedural values (26.8 ± 15.6). This decrease was statistically significant (*P* = 0.013), Table 2 and Figure 2.

## 5. Discussion

VSDs with left ventricular volume overload require closure in order to prevent ventricular dilatation and dysfunction, arrhythmias, aortic regurgitation, pulmonary artery hypertension, and endocarditis [1–18]. Percutaneous closure of VSD is alternative method to surgery in selected cases. This procedure has less negative impact on patient's quality of life; the hospital stay is shorter and causes less pain to surgical treatment. Recent studies in the literature showed that the rate of successful closure has been between 90 and 100% [1–11].

In our study, percutaneous closures of VSDs were successfully performed in 23 patients, and none of the patients need emergency cardiac surgical. The rate of major complications reported in the literature ranges between 0 and 8.6% [1–17]. In our study group, device embolization occurred only in one patient, and the embolized device was rescrewed and removed form the abdominal aorta. VSD in that patient was closed successfully with the same device. Residual shunt was observed in 3 patients after closure. Only in one, residual shunt remained during the followup in whose the defect was closed by ADO II. The most significant early complication after percutaneous VSD closure is atrioventricular block. The complete atrioventricular block rates reported in the literature ranges between 0 and 5.7% [1, 2, 5–13]. In our study complete atrioventricular block was developed in one patient and temporary pacemaker implanted. Block disappeared 24 hours later.

BNP is a neurohormone released by cardiac ventricles in response to volume and pressure load. These natriuretic hormones are thought to be a sensitive and specific indicator of ventricular function [17, 19]. Recent studies have shown that serum BNP level has increased during different types of hemodynamic overload of the heart including VSDs [17, 19]. BNP is correlated with the degree of heart failure and higher in patients with VSDs as a result of hyperkinetic circulation and volume overload. Suda et al. [17] reported that plasma BNP levels significantly positively correlated with pulmonary-to-systemic flow ratio and mean pulmonary artery pressure in children with VSD [4, 5, 12–16]. In our study, we have evaluated the BNP levels one day before and one month after the percutaneous closure. Values of BNP were under the diagnostic limits for overt congestive heart failure. It is possible to explain these BNP values by the absence of overt congestive heart failure and patient's characteristics such as selected small to moderate VSDs with mild pulmonary artery pressure. We observed significant decrease in BNP levels in all patients compared with the values before closure. These findings are thought to be a result of the regression of hypervolemic situation and slowdown of hyperkinetic circulation caused by VSD.

In conclusion, BNP is relatively cheap, noninvasive, rapid, and widely available cardiac biomarker. The current study suggests that serum BNP level may provide a useful clinical tool in evaluation of patients with VSD after the closure. Decrease in serum BNP levels may indicate hemodynamic improvement, so measurement of BNP level may offer an objective method in the clinical evaluation of the patients who underwent percutaneous closure of VSD.

## Figures and Tables

**Table 1:** Patient characteristics and procedures.

Patient no.	Age (yrs)	Gender	ECG	Occluder type	Defect size (mm)	Occluder size (mm)	Number of device	BNP 1	BNP 2
1	8	F	SR	MUSC	10	12	1	94	35
2	34	F	SR	MEMB + ASD	3	4 ADO 2	1	66	12
3	22	F	SR	MUSC	5	6	1	84	26
4	21	F	SR	MUSC	6	8	1	77	30
5	19	M	SR	MEMB + MUSC	8	10	2	65	16
6	53	M	SR	MUSC	6	8	1	303	60
7	38	M	SR	MUSC	5	6	1	45	36
8	24	F	SR	MUSC	8	10	1	86	15
9	9	M	SR	MUSC	5	6	1	56	12

**Table 2:** Summary of procedure data.

Fluoroscopic time (min)	25 ± 20 (range 15–112)		
Procedure time (min)	90 ± 51 (range 40–145)		
Qp/Qs	1.6 ± 0.76 (range 1.2–3.5)		
Systolic PA pressure (mmHg)	48 ± 9		
Mean PA pressure (mmHg)	27 ± 6		
VSD diameter on TTE (mm)	7 ± 2 (range 4–12)		

Types of devices used			
mVSD-O	1 patient		
pmVSD-O	7 patients		
ADO II	1 patient		
Multiple devices	1 patient		

BNP (pg/mL)	Before closure	30 days after closure	*P*
	97.3 ± 78.6	26.8 ± 15.6	0.013
